# An Integrative Approach for Understanding Diversity in the *Punctelia rudecta* Species Complex (Parmeliaceae, Ascomycota)

**DOI:** 10.1371/journal.pone.0146537

**Published:** 2016-02-10

**Authors:** David Alors, H. Thorsten Lumbsch, Pradeep K. Divakar, Steven D. Leavitt, Ana Crespo

**Affiliations:** 1 Departamento de Biología Vegetal II, Facultad de Farmacia, Universidad Complutense de Madrid, Plaza de Ramón y Cajal s/n, Madrid, Spain; 2 Science and Education, Field Museum, Chicago, Illinois, United States of America; Institute of Botany, CHINA

## Abstract

High levels of cryptic diversity have been documented in lichenized fungi, especially in Parmeliaceae, and integrating various lines of evidence, including coalescent-based species delimitation approaches, help establish more robust species circumscriptions. In this study, we used an integrative taxonomic approach to delimit species in the lichen-forming fungal genus *Punctelia* (Parmeliaceae), with a particular focus on the cosmopolitan species *P*. *rudecta*. Nuclear, mitochondrial ribosomal DNA and protein-coding DNA sequences were analyzed in phylogenetic and coalescence-based frameworks. Additionally, morphological, ecological and geographical features of the sampled specimens were evaluated. Five major strongly supported monophyletic clades were recognized in the genus *Punctelia*, and each clade could be characterized by distinct patterns in medullary chemistry. *Punctelia rudecta* as currently circumscribed was shown to be polyphyletic. A variety of empirical species delimitation methods provide evidence for a minimum of four geographically isolated species within the nominal taxon *Punctelia rudecta*, including a newly described saxicolous species, *P*. *guanchica*, and three corticolous species. In order to facilitate reliable sample identification for biodiversity, conservation, and air quality bio-monitoring research, these three species have been epitypified, in addition to the description of a new species.

## Introduction

Circumscribing species in groups with relatively simple and variable morphologies is difficult. Lichen-forming fungi are a prime example of an organismal group where the use of molecular sequence data has challenged many traditional, phenotype-based species circumscriptions. A growing body of evidence indicates that traditionally circumscribed species of lichen-forming fungi commonly mask a substantial amount of species-level diversity [1]. So-called cryptic species with no obvious morphological differences are common in these organisms, especially in Parmeliaceae, one of the largest and most morphologically diverse families of lichenized fungi [1–4]. At least 80 unrecognized species-level lineages have been identified in this family so far [5]. Although some species of lichen-forming fungi have been shown to be truly widespread [6–13], in other cases striking biogeographical patterns have been revealed by revised species delimitation in nominal species that were previously assumed to be cosmopolitan or widely distributed [7,14–20].

An ongoing appeal to researchers to assess species boundaries using multiple lines of evidence (phylogenetics, population genetics, comparative morphology, development, ecology, etc.) has resulted in an increased emphasis to utilize an integrative framework in species delimitation studies [21]. While in practice integrative approaches for species delimitation fall across a broad spectrum, ranging from verbal and qualitative assessments of data classes to quantitative methods that allow different data types to contribute to statistical species delimitation, any study linking different kinds of data to support hypotheses of species boundaries, including placing morphological characters onto a molecular phylogeny, can be considered integrative [22]. Although an iterative process involving the comparison of a molecular phylogeny and morphological data provides a simple approach for species delimitation, integrative taxonomy should combine as many independent lines of evidence as available to reach higher resolution without lack of reliability [23].

*Punctelia* is a medium-sized genus in Parmeliaceae described by Krog [24], with approximately 45 currently accepted species [2]. Species in this genus are characterized by the presence of unciform to filiform conidia, punctiform pseudocyphellae and simple rhizines. The genus has a temperate to subtropical distribution with centers of distribution in the Neotropics and Africa. In a previous study in the genus Punctelia [25], we found evidence that *P*. *rudecta* is not monophyletic, and in this study we focus on delimiting species boundaries in this widespread taxon. *P*. *rudecta* has a subcosmopolitan distribution, being known from North and South America, Africa, and Asia [24,26,27]. In spite of its broad, intercontinental distribution, it appears to be absent from Australasia [28] and records from Europe are dubious [29]. Here we assembled a molecular dataset from *P*. *rudecta* specimens collected from different geographical regions to address its circumscription and test for the potential of geographically isolated, distinct lineages.

The aims of this study are to demonstrate that *Punctelia rudecta* as it is currently circumscribed is poliphyletic and elucidate how many species are masked within this nominal taxon. We collected specimens throughout the known distribution of the species for molecular and morphological evaluation. We focus our efforts on integrating multiple lines of evidence, including molecular phylogenies, coalescent-based species delimitation methods, morphology, ecology, chemistry and biogeography, to assess species circumscription. In addition to evaluating species boundaries within *P*. *rudecta* s. lat., we also provide the most comprehensive phylogeny to date for *Punctelia* genus, including one-third of the known species.

## Materials and Methods

### Taxon sampling

For this study, we sampled a total of 87 *Punctelia* specimens, representing 16 traditionally circumscribed species (S1 Table). *Punctelia* specimens were collected from all continents, with the exception of Antarctica where the species is not known to occur. Overall, our sampling focused on the *P*. *rudecta* s.lat (n = 40) and included specimens from Canada (n = 1), The Canary Islands (10), Chile (2), China (1), India (2), Japan (4), Kenya (5), and USA (15)) (S1 Table). Sequence data was generated from 48 *Punctelia* specimens with vouchers in MAF-Lich and combined with sequences from 39 *Punctelia* specimens downloaded from GenBank (S1 Table). In addition to *Punctelia* samples, *Flavopunctelia flaventior* and *F*. *soredica* were used as outgroups, based on results from previous phylogenetic studies [3,30,31].

All *Punctelia* specimens were examined under a stereomicroscope (Nikon SMZ1000) to study morphological characters (S2 Table). Anatomical characters were studied with a Zeiss Axioscope using hand-cut sections of ascomata and conidiomata. Secondary metabolites were identified by thin-layer chromatography (TLC) following standardized procedures using solvent systems B and C [32].

### DNA extraction and amplification

Small thallus fragments were excised under a dissecting microscope and crushed with sterile glass pestles in liquid nitrogen. Total DNA was extracted using the DNeasy Plant Mini Kit (Qiagen, Hilden, Germany) according to the manufacturer’s instructions, with slight modifications as previously described [33]. We generated sequence data from three loci: the nuclear ribosomal internal transcribed spacer region (nuITS), the mitochondrial small subunit (mtSSU), and the nuclear protein coding locus RNA polymerase II largest subunit (RPB1). PCR amplifications were performed using the following primers: 1) ITS1-F [34] and ITS4 [35] for the nuITS rDNA region; 2) mrSSU1 and mrSSU3 [36] for the mtSSU; and 3) gRPB1-C [37] and RPB1-MH-F [38] for the RPB1. PCR amplifications were conducted in 25 μL with 0.5 μL of Taq polymerase. In some cases where standard PCR failed to amplify target loci, we used Ready-To-Go PCR Beads (GE Healthcare) following manufacturer’s recommendations. PCR amplifications were carried out following conditions: one initial heating step of 4 min at 95°C, followed by 35 cycles of 1 min at 94°C, 1 min at 56°C (ITS), 54°C (mtSSU) and 90 s at 72°C, and a final extension of 10 min at 72°C. The PCR amplification for RPB1 was carried out following the conditions: one initial heating step of 10 min at 94°C, followed by 38 cycles of 45 s at 94°C, 50 s at 56°C and 1 min s at 72°C, and a final extension of 5 min at 72°C.

PCR products were visualized on 1% agarose gel and stained with SYBR safe and cleaned using ExoSAP-IT (USB, Cleveland, Ohio, USA), following manufacturers’ instructions. Complementary strands were sequenced from cleaned PCR products with the same primers used for amplifications. Sequencing reactions were performed using the ABI Prism Dye Terminator Cycle Sequencing Ready Reaction Kit (Applied Biosystems) on a 3730 DNA analyzer (Applied Biosystems) at the Unidad de Genómica (Parque Científico de Madrid).

### Alignments and phylogenetic analyses

Sequences of each locus were aligned using MAFFT 7 [39], with the ‘auto’ mode threshold and default settings. Subsequently ambiguously aligned positions representing were removed using the Gblocks [40] web server (http://molevol.cmima.csic.es/castresana/Gblocks_server.html), implementing the options for a “less stringent” selection of ambiguous regions. Althought Gblocks was observed to worsen the accuracy of phylogenetic tree reconstruction has little inpact under light filtering (up to 20% of aligned positions) [41] we remove 1.3% of aligned positions.Exploratory analyses revealed no significance difference on phylogenetic tree reconstruction.Individual gene alignments were concatenated using Mesquite 2.5 [42]. Exploratory analyses of single-locus phylogenies did not show any well-supported conflicts (bootstrap [14] > 70%) among topologies, and the concatenated dataset was used to infer phylogenetic relationships. Nucleotide substitution models for each locus were chosen using jModeltest [43]: SYM for the ITS marker, HKY85 for mtSSU and K80 for RPB1. These models were specified as imput options in both the Bayesian gene tree inference and the species tree reconstructions. Maximum likelihood (ML) analyses were performed using RAxML 8.1.11 [44] and Bayesian tree samplings were done with MrBayes 3.2 [45]. The ML analysis was performed using the GTRGAMMA model, partitioning the combined 3-marker dataset by locus, and nodal support was assessed using non-parametric bootstrapping carried out for 1000 pseudoreplicates [46]. For the Bayesian tree sampling, the concatenated three-locus data set was partitioned as described in the ML analysis, specifying the best fitting model, and allowing unlinked parameter estimation and independent rate variation. Two parallel runs comprised of 10,000,000 generations, starting with a random tree and employing eight simultaneous chains, were executed. Posterior probabilities (PP) were estimated by sampling trees using a variant of Markov Chain Monte Carlo (MCMC) method. Every 1000^th^ tree was sampled to avoid sample autocorrelation. Based on the likelihood profile, the first 25% trees were discarded as burn in. A 50% majority-rule consensus tree with average branch lengths was computed from the remaining trees, using the sumt option of MrBAYES. Only clades with bootstrap support equal or above 70% under ML and PP equal to or above 0.95 in a Bayesian framework were considered as supported. Phylogenetic trees were visualized using the program FigTree 1.4.0 [47].

### Alternative hypothesis testing

Since *Punctelia rudecta* was recovered as polyphyletic in our analyses (see Results), we tested whether our data is sufficient to reject monophyly of this species as currently circumscribed. For the alternative hypothesis testing, we compared constrained topologies with unconstrained topologies obtained in RaxML (under GTRGAMMA substitution model) using two different methods: i) Shimodaira-Hasegawa (SH) test [48] and ii) expected likelihood weight (ELW) test [49]. The SH and ELW test were performed using Tree-PUZZLE 5.2 [50] with the concatenated data set, comparing the best tree agreeing with the null hypotheses, and the unconstrained ML tree. These trees were evaluated in Tree-PUZZLE using the GTR+I+G nucleotide substitution model. We investigated two constrains to test the polyphyly *of P rudecta* species, ‘constraint 1’ consisted of (*P*. *rudecta* + *P*. *guanchica* + *P*. *ruderata* + *P*. *aff*. *rudecta*). ‘Constraint 2’ consisted of (*P*. *rudecta* + *P*. *guanchica* + *P*. *ruderata* + *P*. *aff*. *rudecta+ P*. *toxodes* + *P*.*missouriensis)* corresponding to species belonging to clades ‘A’ and ‘B’ and excluding the subclade of the sorediate species *P*. *subrudecta* and *P*. *perreticulata*.

### Species delimitation analyses

It has been shown repeatedly that different methods for empirical species delimitation can yield incongruent results (discussed in [51]), which is–among others–due to simplifying assumptions that each of the methods make and different potentials to detect cryptic lineages. Therefore, we explored a variety of methods implemented for both single locus data and other approaches for multilocus sequence data, For the single locus approaches we used the ITS dataset matrix because ITS marker has been adopted as the primary barcode for fungi [52]. Ultimately, we used five different methods to delimit species in the *Punctelia rudecta* group. First, we used the genetic distance-based method “Automatic Barcode Gap Discovery” (ABGD) which takes single locus matrix as input [53]). Two tree-based species delimitation methods–the generalized mixed Yule coalescent model (GMYC) which takes ultrametric single locus as input [54,55] and the Poisson tree process model which takes single locus as input (PTP) [56]–were also used for species delimitation. Finally, two coalescent-based species tree-based methods–species delimitation using species tree using maximum likelihood (spedeSTEM) which takes single locus trees as input, merging all in one to analyze [57] and “Bayesian Phylogenetics and Phylogeography” (BP&P) which takes multilocus data and a prior species tree as input [58]–were implemented to test circumscribe species.

For ABGD we used default parameters except for using a P_max_ at 0.01 and a relative gap width of 0.5. For the GMYC analyses, outgroup samples were excluded from the ITS data set with drop tip in ape [59]. A chronogram was calculated from the ML tree using the penalized likelihood method [60] as implemented in the chronopl command in ape [59,61]. The GMYC method requires a fully dichotomous chronogram and thus we used multdivtime [59] to convert our chronogram into a fully dichotomous chronogram with internal branches of length zero, where appropriate. This modified chronogram was then analyzed using the gmyc function in the SPLITS packagein R (version 2.10, www.cran.r-project.org), employing the single (GMYC_single_) and multiple threshold (GMYC _multiple_) methods. The PTP analysis was done on the web server of the Exelixis Lab (http://species.h-its.org) using default settings. For the spedeSTEM multilocus analysis we used the web server of the Carstens Lab (https://spedestem.osu.edu/runspedestem) with default settings. We used BPP 3.1 [58] which utilizes rjMCMC algorithm and incorporates nearest-neighbor interchange algorithm eliminating the need for a fixed species tree allowing changes in the species tree topologies. To run BPP 3.1 we used the Bayesian species tree performed in *Beast as a prior. The data was analyzed with both algorithm0 and algorithm1 with gamma prior G~ (2, 1000) and the other parameters were assigned to the Dirichlet prior [62] Each analysis was run twice to confirm the consistency between runs and the results showed in this work corresponds to the analysis performed with algorithm1.

### Bayesian species tree

In order to delimit species in a multispecies coalescent framework, we performed a species tree analysis using *Beast as implemented in Beast v.1.8.0 [63,64] and analized in BP&P where the nodal support (e.g., ‘speciation probabilities’) were calculated. The *Punctelia* species tree topology was estimated using a rate-calibrated multilocus coalescent-based species tree approach in *Beast. We used a starting tree based on putative species identified from spedeSTEM, where *P*. *hypoleucites*USA was treated as *P*. *rudecta* s.str. and *P*. *hypoleucites*_from Cuba treated as *P*. *caseana* (see suplementary data). As unique modification we treated *P*. *missouriensis and P*. aff. *rudecta* as single lineages to in order to test this potentially interesting lineages. Using an uncorrelated relaxed lognormal clock [65] we selected a birth-death model for the species tree prior; the population size model was set to piecewise linear and constant root. Molecular evolution rates for the ITS was set at 2.43 × 10^−9^ substitution/site/year (s/s/y) [66] and 1.76 × 10^−9^ s/s/y for RPB1 [67]. Two independent MCMC analyses were run for a total of 100 million generations (sampling every 2000 steps and excluding the first 25 million generations of each run as burn-in). Convergence was assessed by ensuring that standard deviations of split frequencies between runs approached zero, visualizing split probabilities in “Are we there yet?”, AWTY [68] and comparing summarized tree topologies from separate runs. After removing the first 20% of the samples as burn-in, all runs were combined to generate posterior probabilities of nodes from the sampled trees using TreeAnnotator v1.8.0 [69]. Mean node age and 95% highest posterior density (HPD) were mapped on the maximum clade credibility tree. The species tree produced by *BEAST was subsequently used as the guide tree for inferring speciation probabilities in BP&P [70]

### Nomenclature

The electronic version of this article in Portable Document Format (PDF) in a work with an ISSN or ISBN will represent a published work according to the International Code of Nomenclature for algae, fungi, and plants, and hence the new names contained in the electronic publication of a PLOS ONE article are effectively published under that Code from the electronic edition alone, so there is no longer any need to provide printed copies.

In addition, new names contained in this work have been submitted to IndexFungorum from where they will be made available to the Global Names Index. The unique IndexFungorum number can be resolved and the associated information viewed through any standard web browser by appending the IndexFungorum number contained in this publication to the prefix http://www.indexfungorum.org.

## Results

### Phylogenetic analyses and alternative hypothesis testing

**Fig 1:**
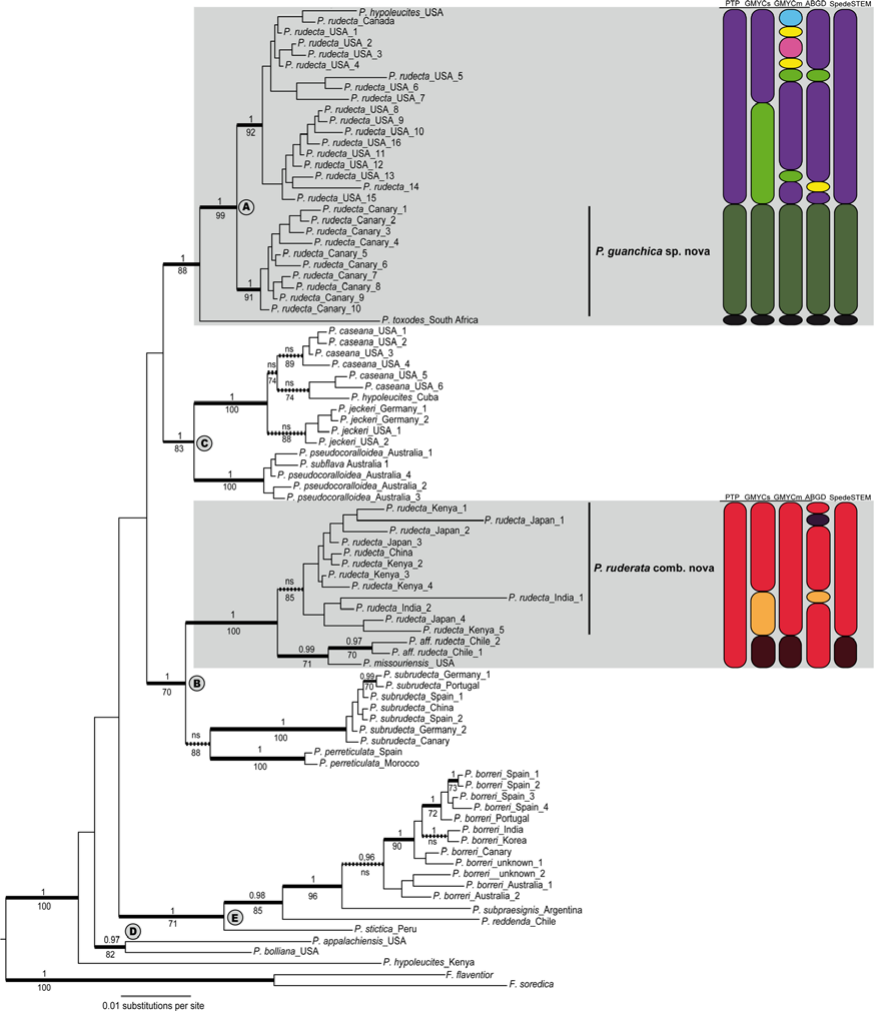
Coalescent-based species tree of the genus *Punctelia*. This is a bayesian species tree inferred in *Beast using three loci (mtSSU, ITS, RPB1). Posterior probabilities are represented by numbers above the branches and nodal support calculated in BP&P with algorithm 1 is indicated with circles on the nodes. Coloured squares represent chemical compounds; species containing gyrophoric acid are shown in yellow and species containing lecanoric acid in gery colours. Abbreviations on the right column are: is = isidiate, sor = sorediate and X = propagules absent.

We assembled a total of 213 DNA sequences from 89 specimens, including 134 new sequences generated for this study (S1 Table). The 476 bp ITS dataset was comprised of 89 sequences, with a nucleotide diversity of 0.043; the mtSSU matrix included 71 sequences (802 bp alignment length), with a nucleotide diversity of 0.019; and the RPB1 data set was comprised of 53 sequences (537 bp), with a nucleotide diversity of 0.020. The concatenated DNA matrix is available online (Available in TreeBase Submision ID-18070). Individual gene topologies didn’t showed well-support topological incongruence (S1, S2 and S3 Figs). Phylogenies inferred from the multilocus, concatenated datasets using ML and Bayesian inference had a similar topology enabling the joint representation (Fig 1). A single difference between the RaxML and Mr.Bayes topologies was the position of three samples “*P*. *hypoleucites_Kenya*”, “*P*. *bolliana_USA*” and “*P*. *appalachiensis_USA*”.

**Fig 2:**
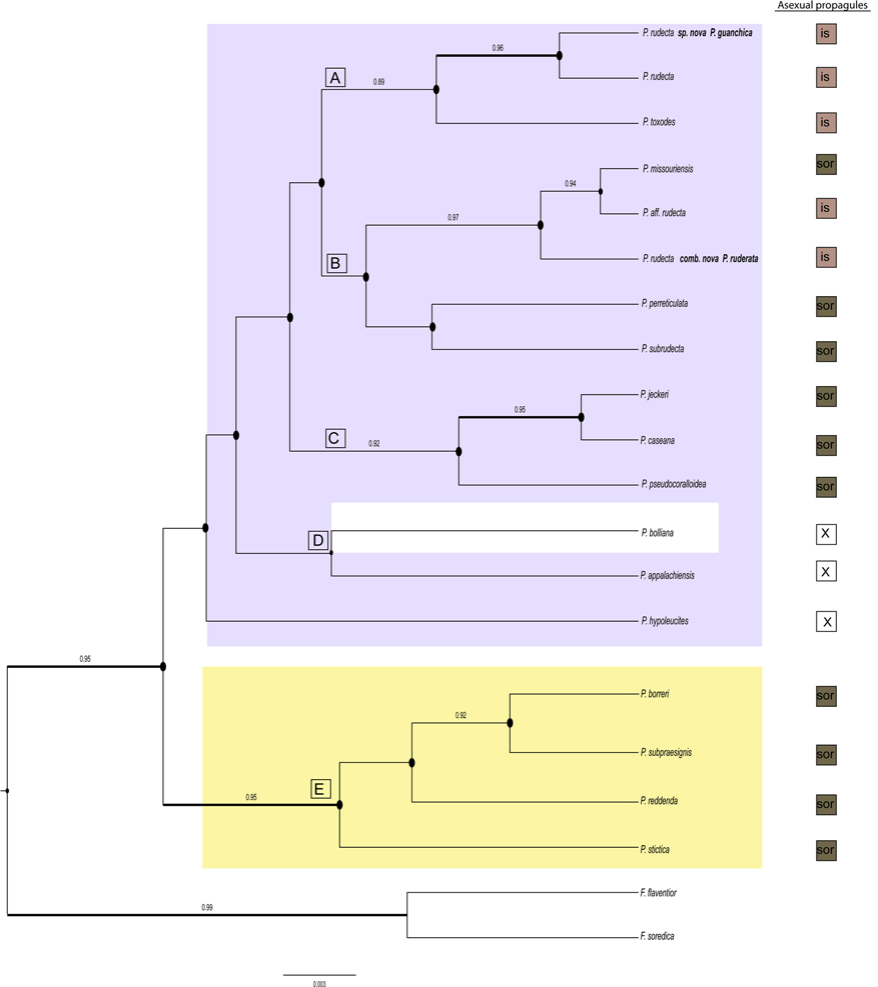
Concatenated gene tree topology. Gene tree of Punctelia genus, focused on *Punctelia rudecta* s. lat. Values on branches represents bootstrap support (bs) equal or higher than 70 and posterior probability (pp) equal or higher than 0.95. Wider bolded lines corresponds to branches supported by both bs and pp, dotted lines represent branches supported by bs or pp. Species delimitation scenarios resulted from different methods indicated in columns to the right (details discussed in text). ns = not supported.

Five major, well-supported clades can be distinguished in the *Punctelia* phylogeney (indicated by the letters A-E). Clade ‘A’–the *P*. *rudecta* group–was comprised of a well-supported clade with samples of *P*. *rudecta* s. lat. from the Canary Islands (hereinafter *P*. *guanchica*), which is sister to a clade that consists of North American *P*. *rudecta* samples (hereinafter *P*. *rudecta* s. stricto), a single specimen identified as *P*. *hypoleucites* downloaded from Genbank, and also a specimen from South Africa identified as *P*. *toxodes* that is sister to the rest of the group. Clade ‘B’ consists of two sister clades. The first is comprised of samples identified as *P*. *rudecta* s. lat. from Asia and East Africa (hereinafter *P*. *ruderata*), and *P*. *missouriensis*, which forms a sister-group to two *P*. aff. *rudecta* samples from Chile. The other group within clade ‘B’ includes two samples of *P*. *perreticulata* and seven samples of *P*. *subrudecta*. Both these species form well-supported monophyletic lineages. Clade ‘C’ includes two well-supported clades, one with samples representing *P*. *caseana* and *P*. *jeckeri* (each forming monophyletic groups, but only the latter being strongly supported), whereas the other clade includes samples from Australia that were identified as *P*. *pseudocoralloidea* and *P*. *subflava*. The species of clades ‘A’-‘C’ are all characterized by the presence of lecanoric acid as major medullary component and forms a well-supported clade in trees performed in *Beast species tree (Fig 2). Clade ‘D’ includes samples of two species containing fatty acids as major medullary compounds, *P*. *appalachensis* and *P*. *bolliana*. Clade ‘E’ includes species containing gyrophoric acid as main compound and is comprised of samples representing *P*. *borreri*, forming a well-supported monophyletic group, nested to *P*. *subpraesignis*,*P*. *reddenda*, and *P*. *stictica*. The relationship of *P*. *hypoleucites* from Kenya is uncertain and was recovered as basal to all the clades (Fig 1).

*Punctelia rudecta* as currently circumscribed was recovered as polyphyletic with samples forming distinct lineages within clades ‘A’ and ‘B’ (Fig 2), with each lineages restricted to distinct geographic regions. Chilean samples representing *P*. *rudecta* s. lat. formed a monophyletic group within clade ‘B’, specimens representing *P*. *ruderata* s. lat. from Africa and Asia formed another monophyletic group within clade ‘B’, and North American material representing *P*. *rudecta* s. str. were restricted to a strongly supported group within clade ‘A’. Finally, all *P*. *rudecta* s. lat. specimens from the Canary Islands formed a sister-group to the North American specimens within clade ‘A’. A single collection representing *P*. *toxodes* from South Africa was sister to all remaining samples within clade ‘A’. Alternative hypothesis tests rejected monophyly of *P*. *rudecta* under both tests (SH and ELH); however, only ‘constraint 2’ strongly rejects monophyly of *P*. *rudecta s*. *lat*. (p<0.001 in SH and ELW tests) (Table 1).

**Table 1:** Alternative hypothesis testing.

Alternative hypothesis testing	SH p-value	ELH p-value
Constrain 1	0.047*	0.0477*
Constrain 2	< 0.001***	< 0.001***

Asterisk symbols represents the level of statistical significance.

*, p value equal or less than 0.05.

***, p value equal or less than 0.001.

### Species delimitation analyses

The sampling for the species delimitation analyses was only sufficient for *P*. *rudecta* s. lat. and hence all other *Punctelia* species included in this study are assumed to represent the identified nominal taxa. Speciation probabilities estimated in BP&P shown high PP for most of the candidate species in the *Punctelia rudecta* complex, including: *P*. *guanchica*, *P*. *rudecta* s. stricto, *P*. *toxodes* and *P*. *ruderata* (Fig 2). However, the isidiate samples identified as *P*. *rudecta* from Chile or the sorediate *P*. *missouriensis* were not supported with high speciation probabilities (Fig 2). The species tree inferred using *Beast showed high PP for the sorediate species included in the A-B clade *P*. *perreticulata* and *P*. *subrudecta* included in clade B. For *P*. *rudecta* specimens recovered in clade ‘A’, the additional analyses (ABGD, GMYC_single_, GMYC_multiple_, PTP, spedeSTEM) supported the clade consisting of *P*. *guanchica* samples from the Canary Islands as a distinct, species-level lineage (Fig 1, Fig 2). The results for its sister-group, *P*. *rudecta* s. stricto from North America is a well supported lineage but is recognized by GMYC_single_ as two different lineages, while ABGD and GMYC_multiple_, recognized additional putative lineages within this group (Fig 1). The South African sample recognized as *P*. *toxodes*, was identified as a distinct lineage in all six analyses.

In clade ‘B’, *P*. *ruderata*, *P*. *aff*. *rudecta* and *P*. *missouriensis* were included into a single lineage by PTP, while GMYC_single_, GMYC_multiple_ and spedeSTEM recognized one lineage composed by *P*. *missouriensis* and *P*. *aff*. *rudecta* differentiated from P., whereas ABGD identified multiple lineages (Fig 1).

### Phenotypical re-examination of *Punctelia rudecta* samples

In order to phenotypically characterize putative species-level lineages found in our sampling of isidiate specimens in clades ‘A’ and ‘B’ (*P*. *rudecta* sampling s. lat.), we studied morphological characters and secondary chemistry of the samples included in the analyses. Morphological characters, such as thallus color, presence and form of isidia, presence of ascomata, and conidiomata, showed some variability but only few of these characters were associated with the clades found in the phylogenetic analyses (S1 Table). Thallus color was variable within putative lineages, and failed to consistently diagnose species-level lineages. The lower surface was usually pale brown, but also ranging into a darker brown coloration in some specimens. The upper surface showed a gradation from brownish green to bluish grey. To ensure any result about the importance of color, only recently collected specimens were considered because the color tonality is gradually lost after collection. Isidia morphology showed some variability even among lobes within the same thallus. In order to systematize the isidia form comparison, the degree of ramification was taken in account, and two times dichotomous ramificated isidia were most commonly observed. When the isidia were three or more times dichotomously branched, they took coralloid or dendroid appearance. The specimen representing *P*. *toxodes* was the only collection showing unbranched isidia. Despite the inclusion of a number of collections from the Canary Islands, we failed to find ascomata or conidiomata on them, whereas specimens from East Africa, Asia or South America frequently have mature ascomata. In North American samples, young immature ascomata were occasionally found. Ascospores comparison between groups was not possible, and a comparison of conidiospore size failed to distinguish distinct lineages. It is interesting to note that in *P*. *ruderata* and *P*. *aff*. *rudecta* from Chile conidiomata and ascomata were found within the same thallus usually however these are not found in the single thallus of the sample of *P*. *rudecta* or *P*. *toxodes*. While all specimens of *P*. *rudecta* s. lat. contained atranorin and lecanoric acid as major compounds, the samples from Chile, Kenya, Japan and India often also contained unidentified fatty acids, which were not found in the other specimens examined. While most *P*. *rudecta* s. lat. species occur mainly on bark, *P*. *guanchica* sp. nov. differs in substrate preference, occurring mainly rocks and occasionally the base of a shrub. We have confirmation that the species *P*. *toxodes* also occurs on rock (personal observation).

## Discussion

### Phylogenetic analyses and species delimitation

Inferences from previous phylogenetic studies on *Punctelia* have been limited by relatively small sample sizes and the use of only one or two molecular markers [25,71]. In this study, five major, well-supported clades were found in *Punctelia* (S2 Fig), although relationships among these clades were partially known previously (Fig 2). Interestingly the major clades in *Punctelia* are characterized by their medullary chemistry. Clades ‘A’, ‘B’, and ‘C’ are comprised of specimens producing the depside lecanoric acid, whereas species in clade ‘D’ produce the related tridepside gyrophoric acid. Clade ‘E’ is characterized the presence of fatty acids. Secondary chemistry has been found previously in other, only distantly related groups of lichenized fungi to be a predictor of phylogenetic relationships, including Baeomycetaceae [72], Graphidaceae [73], Pertusariaceae [74,75], Teloschistaceae [76], and also several clades within Parmeliaceae [3,77,78].

*Punctelia rudecta* as currently circumscribed has been shown to be polyphyletic with samples falling into five distinct clades. The BP&P analysis showed species with high statistical support in the isidiate species complex belonging to clades ‘A’ and ‘B’ (Fig 2) recognizing four species; *P*. *rudecta*, *P*. *guanchica* sp. nov., *P*. *toxodes*, and *P*. *ruderata*. Nodal support for the *P*. *guanchica*-*P*.*rudecta* 1 clade, the node separating *P*. *toxodes* from the previous node 1, the node which separates *P*. *ruderata* from *P*. *missouriensis*, and *P*. *aff*. *rudecta* were all estimated at PP = 0.99 (Fig 2). The node separating *P*. *missouriensis* and *P*. *aff rudecta* was the only split that didn’t showed strong statistical support, and it appears that this uncertainty most probably results from the fact that this lineages was represented only by ITS sequence data (S1 Table).

Species delimitation analyses based on additional analyses supported between four and ten candidate species in *P*. *rudecta* s. lat. (Fig 1), with PTP identifying the lowest (4 OTUs) and GMYC_multiple_ the highest number of species (10 OTUs). The latter method has been suggested to overestimate the number of clusters under certain conditions [79,80]. With the data at hand, we follow a conservative approach as advocated by Miralles and Vences [23]who argued that it would be better to fail to delimit species to falsely delimit entities that do not represent independent lineages. The results of PTP, SPedeSTEM and BPP were consistent in circumscribing four species in both the PTP and BP&P analysis, plus a fifth lineage supported in the SpedeSTEM analysis which included the samples *P*. *missouriensis* and *P*. *aff*. *rudecta* (Fig 1, Fig 2). The taxonomic conclusions to accommodate the four species identified under the name *Punctelia rudecta* are drawn in the section below.

This study adds another example to the growing number of cases reviewed [5,22,81,82]in which species that were thought to have wide distributional ranges have subsequently been split into distinct cryptic lineages with more restricted distributional ranges,. Morphological and chemical reexamination of the samples did not reveal phenotypical characters that would allow identification without having molecular data at hand, except the presence of unidentified fatty acids in *P*. *ruderata* and *P*. *aff*. *rudecta*, and smaller and unbranched isidia in the South African material named *Punctelia toxodes*. This name has recently been resurrected based on morphological features, including dorsiventrally flattened isidia and unciform, cylindrical, curved conidia [83]. In our samples the isidia are shorter, unbranched, thick and rarely flattened with age. Since we only had two specimens from South Africa available for study and were unable to assess the variation of these characters within the population. Isidial morphology is very variable in *Punctelia rudecta* sensu lato and in lichenized fungi in general and thus it could not be considered as a useful feature to discriminate species. Ecological traits can be useful to discriminate *P*. *guanchica* sp. nov. which grows on volcanic rocks, while our samples of *P*. *rudecta* s. stricto, *P*. *aff*. *rudecta* and *P*. *ruderata* were exclusively corticolous. The taxon *P*. *toxodes* is known to grow on both rocks and tree bark. The identification of specimens will not cause issues for field biologists, since the species that are here recognized are geographically isolated. Geographical structure of species identified using molecular data has been recently shown to be a common phenomenon in lichenized fungi [14,15,18–20].

Here we use an integrative taxonomic approach to circumscribe and formally recognize species in *P*. *rudecta* s. lat. Integrative taxonomy uses independent sources of data, such as molecular, morphological, ecological, and/or geographical data to delimit species [84]. However, these data sources are limited especially for lichenized and non-lichenized fungi and collecting these could be a great challenge for cryptic lineages. In these cases, coalescent-based approaches have been widely used as an objective measure for identifying divergent evolutionary lineages [21,51]. Nonetheless, identifying geographical/and or ecological differences between lineages could also help to understand speciation process. Our results show that implementing a variety of species delimitation methods, coupled with assessment of other data types–as in this case geography–could play an important role in identification of species and speciation process in lichenized fungi.

### Taxonomic conclusions

As discussed above, we propose the formal recognition of four species in the *Punctelia rudecta* s. lat: *P*. *rudecta* s. str., *P*. *ruderata*, *P*. *toxodes*, and the newly described *P*. *guanchica* sp. nov. The type specimen of *P*. *rudecta* was collected in North America [27,85], and hence this name should be used for the North American clade (epitype: USA, North Carolina, Swain County, Great Smoky Mountains National Park, Beauregard Ridge, 35°27’38”N, 83°26’17”W, alt. 560m, On *Acer*, 14^th^ Oct. 2010, *J*.*C*. *Lendemer* 26959 [MAF-Lich 19163; Ref. Sequences KR024454 (ITS), KR024500 (mtSSU), KR024541 (RPB1)], selected here). The name *Punctelia toxodes* (Stirt.) Kalb & Götz. (Bas.: *Parmelia toxodes* Stirt., Scot. Nat. 4: 253 [1877–78]; epitype: South Africa, Cape region, Paarl Mountain, 33°44’26”S, 18°56’55”E, alt. 530m, on tree trunk and twigs, 30^th^ May 2005, *A*. *Crespo*, *P*.*K*. *Divakar*, *D*.*L*. *Hawksworth*, *G*. *Amo & H*.*T*. *Lumbsch* [MAF-Lich 19757; Ref. Sequences KR024412 (ITS), KR024460 (mtSSU), KR024504 (RPB1)]), selected here) is available for the South African clade. The name *Punctelia ruderata* (Vainio) Canêz & Marcelli was resurrected in a Ph.D. thesis [86] and reported from Brazil. However, the combination has not been validly published (Art 29.1, ICBN). Further, our results indicate that *P*. *ruderata* in our circumscription does not occur in Brazil. Therefore, for the Old world clade identified in this study, we propose to resurrect the name ***Punctelia ruderata* (Vain.)** Canêz & Marcelli ex. **Alors et al. comb nov.** (Index Fungorum no. IF 551455; Bas.: *Parmelia ruderata* Vain., Bot. Mag. Tokyo 35: 47 [1921]; epitype: Japan, Honshu, Musashi (Pref. Saitama), Takizawa Dam, Ohtaki, Chichibu city, 35° 56’ 31”N 138° 53’ 58”E alt. 577m, on *Prunus yedoensis*, 7^th^ Feb. 2009, *A*. *Crespo*, *P*.*K*. *Divakar & Y*. *Ohmura* 6018w [MAF-Lich 18877; Ref. Sequences KR024434 (ITS), KR024521 (RPB1)]), selected here). There is no available name for the Canary Islands clade and hence we describe a new species for this clade below.

***Punctelia guanchica* Alors**, **A**. **Crespo & Divakar**, **sp**. **nov**.

Index Fungorum: IF551458

*Diagnosis*: Morphologically similar to *P*. *rudecta* sensu stricto, but differs in having thicker isidia developing from the centre of pseudocyphellae, saxicolous habitat, and DNA characters; only known from the Canary Islands.

*Type*: Spain: Canary Islands, Tenerife, La laguna, Las Mercedes, monte verde, 28°32’ 41”N 16°17’49”W, alt. 613m, on vertical basaltic rock, 20^th^ June 2009, *A*. *Crespo*,*P*. *Cubas*, *A*. *Santos & P*. *K*. *Divakar*, 6046v (MAF-Lich 18871−holotype; F−isotype).

**Fig 3:**
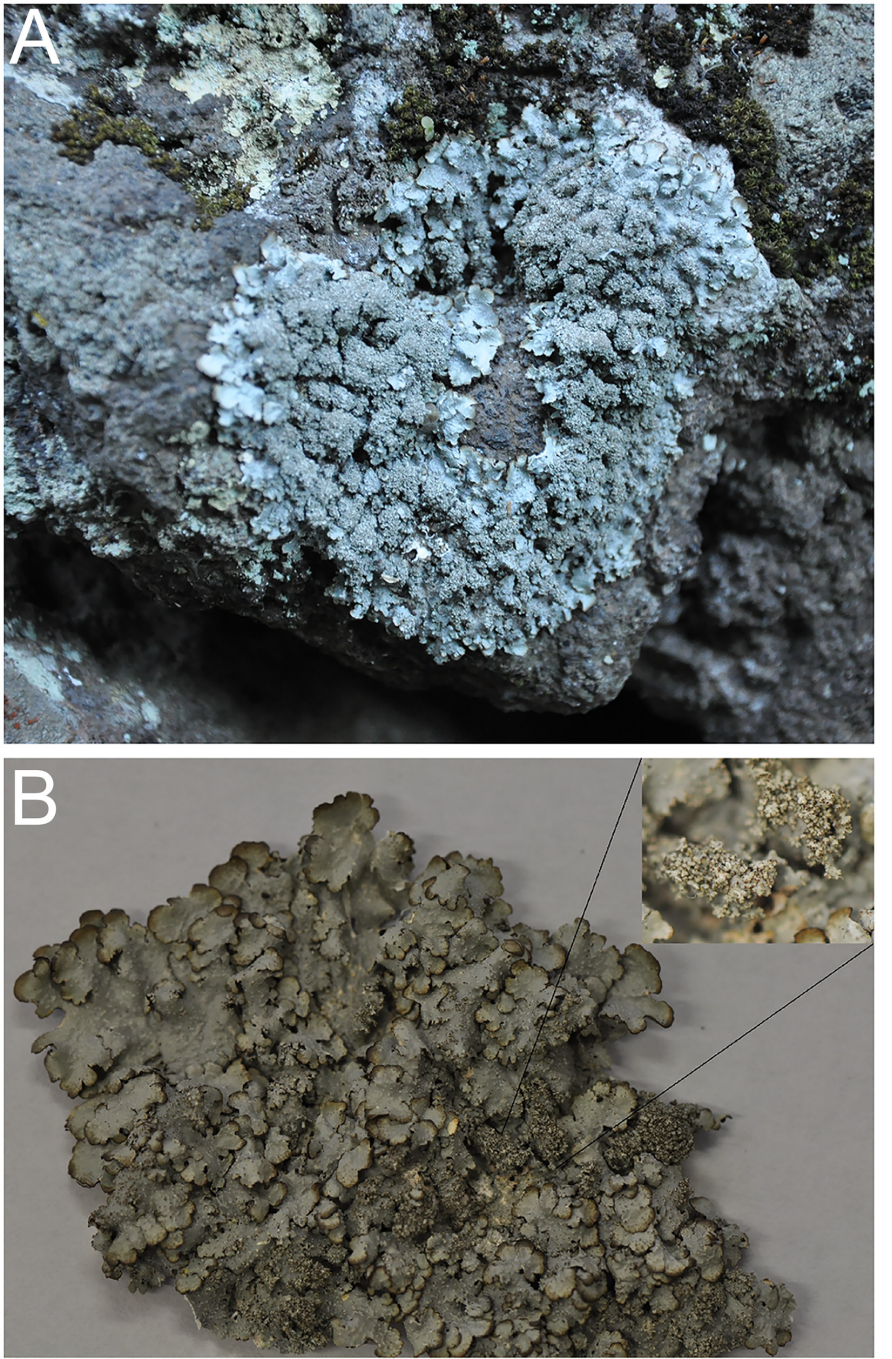
*Punctelia guanchica*. (A) showing habitat of Punctelia *guanchica*, (B) habit of *P*. *guanchica* with enhanced detail of isidia in the right upper corner of the picture.

*Description*: Thallus foliose, adpressed to the substratum, 3–4 cm across; lobes rotund, 2–4 mm wide, margins entire, eciliate. Upper surface whitish grey, often bordered by a narrow, brown margin, reticulately rugulose near the margin of lobes, pseudocyphellate and isidiate. Pseudocyphellae laminal, punctiform to elongate, up to 0.5 mm in size, more distinct near peripheral zone. Isidia laminal, developing from the centre of pseudocyphellae, thick, short, up to 0.5 mm tall, simple to coralloid branched, in groups, rarely flattened in the centre of thallus (Fig 3). Medulla white. Lower surface white, with pale margin, rhizines simple, concolours with the lower surface, ca. 1 mm long. Photobiont trebouxioid. Apothecia and conidia unknown.

*Chemistry*: Cortex K+ yellow; medulla K-, C+ rose, KC+ red, PD-; atranorin and lecanoric acid.

*Reference sequences*: GenBank accession numbers. KR024415 (ITS), KR024463 (mtSSU) and KR024506 (RPB1)

*Additional specimens examined*: The samples are listed in S1 Table.

*Etymology*: The epithet ‘*guanchica*’ refers to the guanches, the aboriginal inhabitants of the Canary Islands, since the species is only known from this archipelago.

*Remarks*: In the field the new species can easily be confused with *P*. *rudecta* sensu stricto, which occurs in North America and differs in having isidia developing from the periphery of pseudocyphellae, and is mainly corticolous. *P*. *guanchica* is only known from Canary Islands and has only been found on saxicolous habitats. The new species is also similar to *P*. *toxodes* that is endemic to South Africa. Morphologically *P*. *guanchica* is also similar to *P*. *ruderata*, which occurs in Asia and East Africa, and belongs to an independent, supported monophyletic clade (clade B, Fig 1 Fig 2).

## Supporting Information

S1 Fig ITS ML tree. ML phylogenetic tree of Punctelia genus from ITS sequences. Bolded lines represent supported branches with bootstrap values higher or equal than 70.
(PDF)

S2 Fig mtSSU tree. ML phylogenetic tree of Punctelia genus from mtSSU sequences. Bolded lines represent supported branches with bootstrap values higher or equal than 70.
(PDF)

S3 Fig RPB1 tree. ML phylogenetic tree of Punctelia genus from RPB1 sequences. Bolded lines represent supported branches with bootstrap values higher or equal than 70.
(PDF)

S1 Table Collection information for all the specimens included in the present study. GenBank accession numbers for the three sampled loci: nuclear ribosomal internal transcribed spacer region (ITS), mitochondrial small subunit (mtSSU), and protein-coding maker RPB1. Newly generated sequences for this study are indicated in boldface. Type specimens are indicated with asterisk (*).
(XLS)

S2 Table Specimens re-examined. Table summarizing information obtained from the vouchers of *P*. *rudecta* s. lat. samples, including size, presence of sexual structures, coloration, chemistry and substrate.
(XLSX)
