# Supplementary figures and images for: An Integrative Approach for Understanding Diversity in the Punctelia rudecta Species Complex (Parmeliaceae, Ascomycota)
Source: PLoS One. 2016 Feb 10;11(2):e0146537. doi: 10.1371/journal.pone.0146537 (PMC4749632; doi:10.1371/journal.pone.0146537)

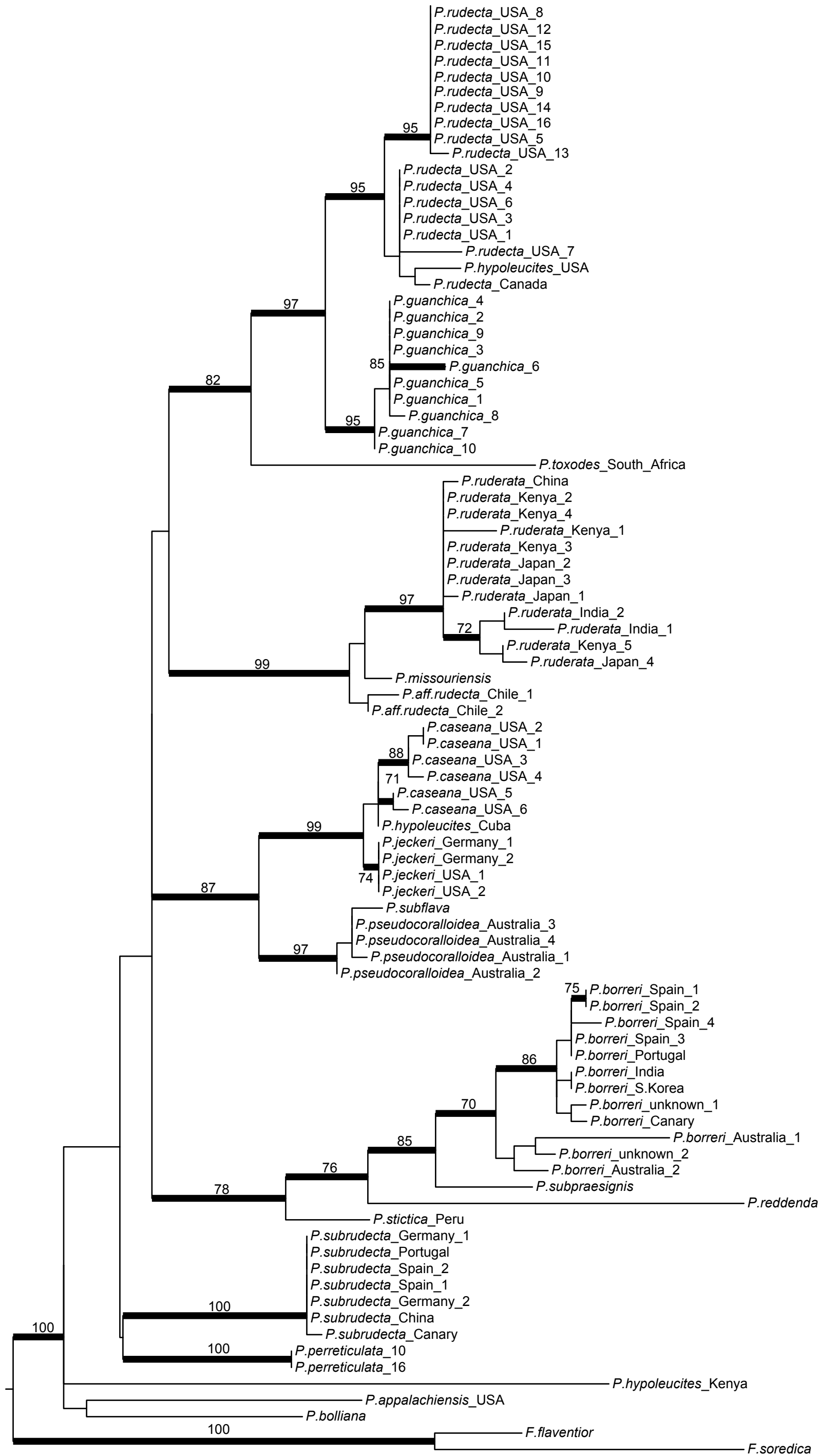

0.02 substitutions per site

Supplement: S1 Fig — ML phylogenetic tree of Punctelia genus from ITS sequences. Bolded lines represent supported branches with bootstrap values higher or equal than 70. (PDF) [file pone.0146537.s001.pdf]

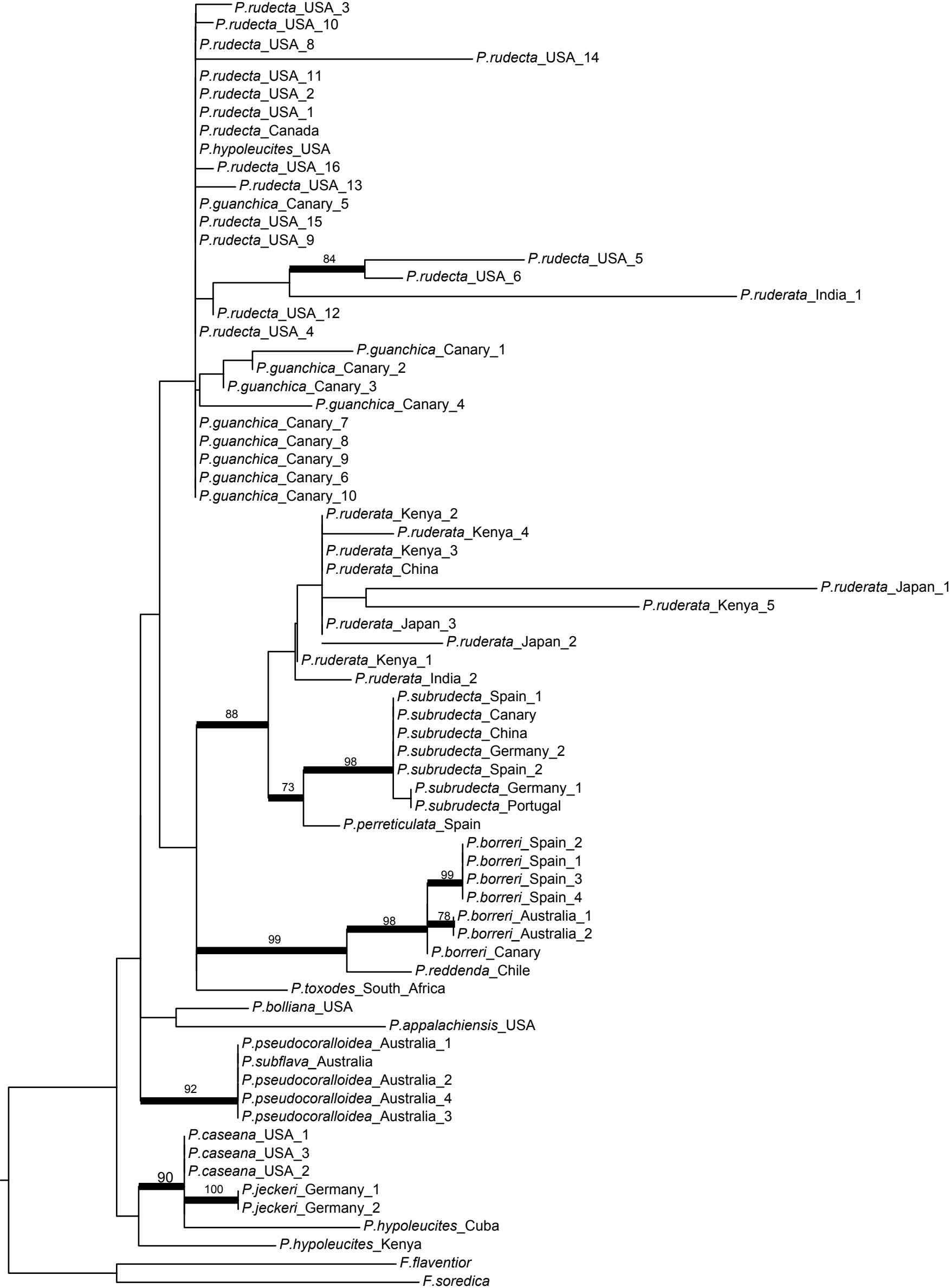

0.006 substitution per site

Supplement: S2 Fig — ML phylogenetic tree of Punctelia genus from mtSSU sequences. Bolded lines represent supported branches with bootstrap values higher or equal than 70. (PDF) [file pone.0146537.s002.pdf]

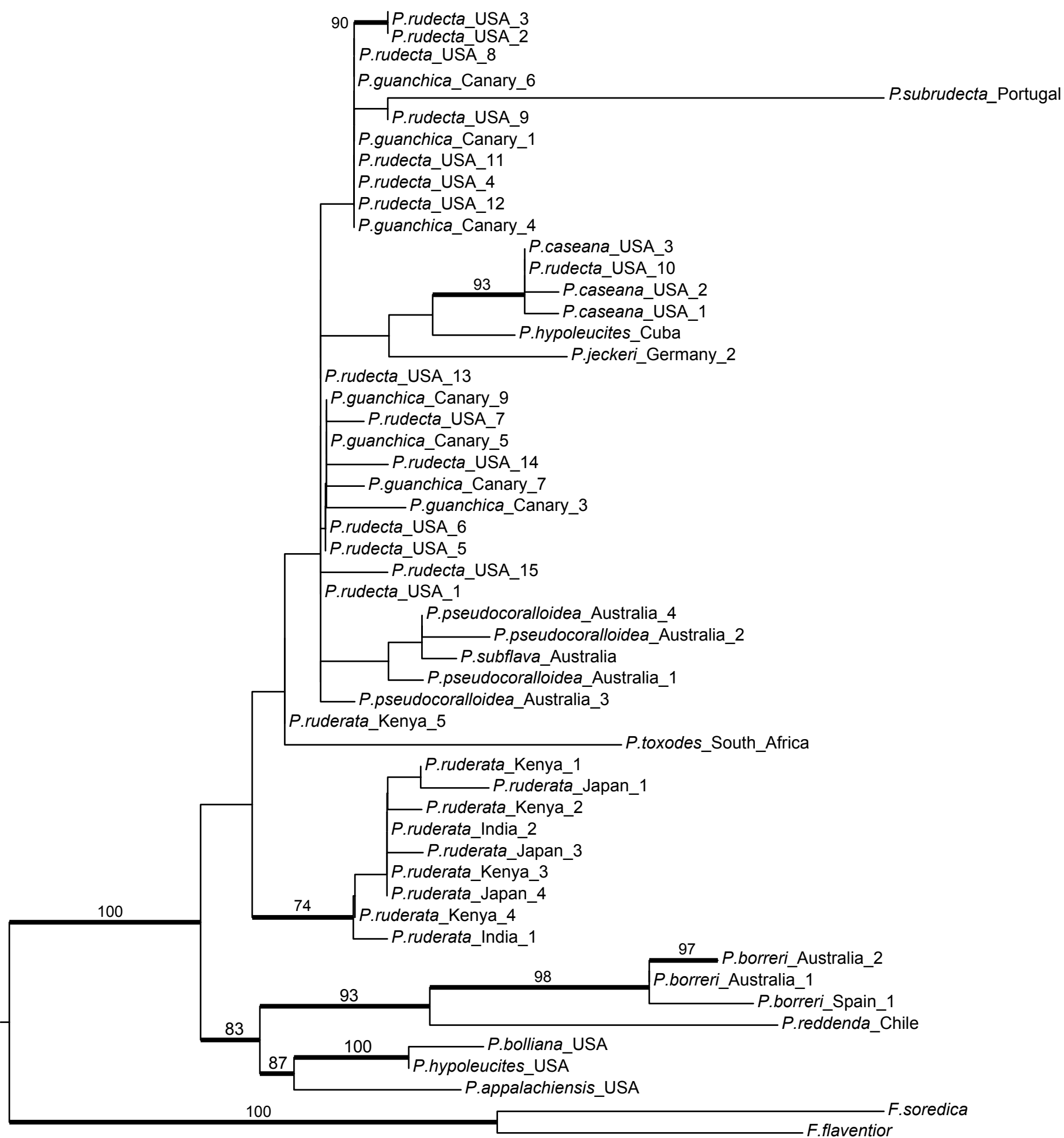

0.006 substitutions per site

Supplement: S3 Fig — ML phylogenetic tree of Punctelia genus from RPB1 sequences. Bolded lines represent supported branches with bootstrap values higher or equal than 70. (PDF) [file pone.0146537.s003.pdf]
